# Impact of Emergency Department-Initiated Buprenorphine on Repeat Emergency Department Utilization

**DOI:** 10.5811/westjem.60511

**Published:** 2023-11-08

**Authors:** Rachel M. Skains, Lindy Reynolds, Nicholas Carlisle, Sonya Heath, Whitney Covington, Kyle Hornbuckle, Lauren Walter

**Affiliations:** * University of Alabama at Birmingham, Heersink School of Medicine, Department of Emergency Medicine, Birmingham, Alabama; † Birmingham VA Medical Center, Department of Emergency Medicine, Birmingham, Alabama; ‡ University of Alabama at Birmingham, School of Public Health, Department of Health Behavior, Birmingham, Alabama; § University of Alabama at Birmingham, Heersink School of Medicine, Department of Internal Medicine, Birmingham, Alabama; ∥ University of Alabama at Birmingham, Heersink School of Medicine, Birmingham, Alabama

## Abstract

**Introduction:**

Recent studies have demonstrated the promise of emergency department (ED)-initiated buprenorphine/naloxone (bup/nx) for improving 30-day retention in outpatient addiction care programs for patients with opioid use disorder (OUD). We investigated whether ED-initiated bup/nx for OUD also impacts repeat ED utilization.

**Methods:**

We performed a retrospective chart review of ED patients discharged with a primary diagnosis of OUD from July 2019–December 2020. Characteristics considered included age, gender, race, insurance status, domicile status, presence of comorbid Diagnostic and Statistical Manual of Mental Disorders, Fifth Edition (DSM-5) diagnosis, presenting chief complaint, and provision of a bup/nx prescription and/or naloxone kit. Primary outcomes included repeat ED visit (opioid or non-opioid related) within 30 days, 90 days, and one year. Statistical analyses included bivariate comparison and Poisson regression.

**Results:**

Of 169 participants, the majority were male (67.5%), White (82.8%), uninsured (72.2%), and in opioid withdrawal and/or requesting “detox” (75.7%). Ninety-one (53.8%) received ED-initiated bup/nx, which was independent of age, gender, race, insurance status, presence of comorbid DSM-5 diagnosis, or domicile status. Naloxone was more likely to be provided to patients who received bup/nx (97.8% vs 26.9%; *P* < 0.001), and bup/nx was more likely to be given to patients who presented with opioid withdrawal and/or requested “detox” (63.3% vs 36.7%; *P* < 0.001). Bup/nx provision was associated with decreased ED utilization for opioid-related visits at 30 days (*P* = 0.04). Homelessness and lack of insurance were associated with increased ED utilization for non-opioid-related visits at 90 days (*P* = 0.008 and *P* = 0.005, respectively), and again at one year for homelessness (*P* < 0.001). When controlling for age and domicile status, the adjusted incidence rate ratio for overall ED visits was 0.56 (95% confidence interval [CI] 0.33–0.96) at 30 days, 0.43 (95% CI 0.27–0.69) at 90 days, and 0.60 (95% CI 0.39–0.92) at one year, favoring bup/nx provision.

**Conclusion:**

Initiation of bup/nx in the ED setting was associated with decreased subsequent ED utilization. Socioeconomic factors, specifically health insurance and domicile status, significantly impacted non-opioid-related ED reuse. These findings demonstrate the ED’s potential as an initiation point for bup/nx and highlight the importance of considering the social risk and social need for OUD patients.

Population Health Research Capsule What do we already know about this issue?

*Emergency department (ED)-initiated buprenorphine/naloxone (bup/nx) improves 30-day retention in outpatient addiction programs for opioid use disorder (OUD).*

What was the research question?

*Does ED-initiated bup/nx for OUD also impact acute healthcare utilization, specifically repeat ED visits, for OUD patients?*

What was the major quantitative finding of the study?

*Bup/nx decreased ED utilization at 30 days (37.5% vs. 62.5%, P < 0.05). Homelessness and lack of insurance increased ED utilization at 90 days (P < 0.01).*

How does this improve population health?

*Findings show the ED’s potential as an initiation point for bup/nx and highlight the importance of social risk and need for OUD patients.*

## INTRODUCTION

According to the Substance Abuse and Mental Health Services Administration, nearly 5.6 million residents of the United States had opioid use disorder (OUD) in 2021, accounting for 2% of the US population.^1^ From 2020 to 2021, there were an estimated 1.8 million new users of prescription pain relievers and 26,000 new heroin users, or nearly 5,000 new opioid users per day.^1^ Correspondingly, the US Centers for Disease Control and Prevention observed a record high drug overdose mortality in 2021, with over 107,000 drug overdose deaths in the US, more than 80,000 of which involved opioids.^2^

The state of Alabama has been particularly affected by the opioid epidemic. Since 2014, Alabama has led the nation with the highest rate of opioid prescriptions in the country (80.4 prescriptions for every 100 persons in 2020), approximately twofold greater than the national average.^3^ Jefferson County, the state’s most populous county, had the highest number of opioid overdose deaths in Alabama in 2021, with 342 confirmed opioid overdose deaths, a 44.7% increase from 2020.^4^ The opioid epidemic is an ongoing, significant public health emergency as evidenced by the rising incidence of opioid misuse, OUD, and opioid-related deaths in the US.

Emergency physicians are uniquely positioned to help combat the growing opioid crisis by screening and initiating care for patients presenting to the emergency department (ED) with OUD. Opioid-related ED visits have increased, representing nearly one in 80 ED visits, and escalated dramatically during the coronavirus disease 2019 (COVID-19) pandemic when non-opioid-related ED visits decreased.^5^ ^,^ ^6^ Importantly, screening for opioid misuse and dependence in the ED has been proven to positively affect the prognosis of these patients. In a landmark randomized clinical trial in 2015, D’Onofrio and colleagues demonstrated that ED screening, brief intervention, and referral to treatment (SBIRT) for OUD, including ED-initiated medications for OUD (MOUD) with buprenorphine/naloxone (bup/nx), significantly increased 30-day retention in outpatient addiction treatment, decreased the use of opioids, and decreased utilization of inpatient addiction services.^7^ As MOUD has been recognized as an effective treatment option to reduce mortality, overdose, and cost, EDs are increasingly engaged in OUD treatment initiation.^8^ ^–^ ^14^ Further, a recent community-based study by Le et al demonstrated decreased subsequent healthcare utilization at 12 months after initiation of MOUD in the ED.^15^

Most ED-initiated MOUD studies have focused on treatment retention in large, urban, academic medical centers outside the Southeast or subsequent healthcare utilization in community hospitals.^7^ ^,^ ^11^ ^,^ ^12^ ^,^ ^15^ ^,^ ^16^ Our large, urban, academic ED in the Southeast offers a unique perspective on the impact of ED-initiated MOUD on healthcare utilization in a resource-limited region characterized by persistent Medicaid non-expansion, high poverty rates, and healthcare access challenges.^13^ In this study, we investigated whether ED-initiated bup/nx also impacts acute healthcare utilization, specifically repeat ED visits, for ED OUD patients.

## METHODS

### Study Design and Setting

We conducted a retrospective chart review of patients who presented to our urban academic medical center ED at the University of Alabama at Birmingham (UAB) and were discharged from the ED with a diagnosis of OUD, using International Classification of Diseases, 10^th^ Revision, (ICD-10) code documentation.^17^ We obtained UAB Institutional Review Board approval. Our 48-bed, tertiary care ED evaluates over 75,000 patients annually. The UAB Hospital has 1,157 licensed beds and serves as the primary hospital for north-central Alabama and surrounding areas. We selected the study period July 2019–June 2020 because it marked the inaugural year of the hospital’s ED-initiated OUD program, where patients with a diagnosis of OUD were to be discharged with a bridge bup/nx prescription, naloxone take-home kit, and referral to outpatient addiction treatment. However, emergency clinicians’ uptake and utilization of the bup/nx prescription was not universal during that first year. Prior to July 2019, bup/nx was not routinely prescribed from the ED.

### Study Variables

The primary outcomes of interest were repeat ED utilization within 30 days, 90 days, and one year of the initial ED visit. Repeat ED visits were further classified as either opioid-related or non-opioid-related, as defined by ICD-10 documentation.^17^ When analyzing opioid-related ED visits and non-opioid-related ED visits separately, we considered outcomes at each time point as binary variables. The number of opioid-related repeat ED visits was added to the number of non-opioid-related ED visits within 30 days, 90 days, and one year to obtain the composite outcome of total repeat ED visits at each time point of interest. We used composite value for Poisson regression analysis. The primary exposure of interest was whether the patient was discharged with a bup/nx prescription, which was a binary variable coded as yes or no.

Other variables in the analysis included age, gender, race, health insurance status, domicile status, provision of a naloxone kit, comorbid Diagnostic and Statistical Manual of Mental Disorders, Fifth Edition (DSM-5) diagnosis, and presenting chief complaint at the initial ED visit. Age was measured in years and was examined as a continuous variable. Gender was determined by data recorded in the electronic health record (EHR) at the time of ED registration, typically dictated by available legal identification (eg, driver’s license) or self-reported in absence of ID. Gender was a nominal variable classified as male, female or other, per EHR limitations. Race was categorized as White or Black. (Other racial categories were not considered due to low numbers.) Health insurance was defined as private, public (Medicare and/or Medicaid), or self-pay (uninsured). Domicile status was a binary variable and classified as either homeless or not homeless. The provision of a naloxone kit upon discharge from initial visit was included as a dichotomous yes or no variable, as was the presence of a comorbid DSM-5 mental health diagnosis. Concomitant mental health diagnosis was determined by presence in “past medical history” during chart review. Chief complaint at the initial ED visit was noted and was manually classified by reviewers as opioid withdrawal/detoxification (“detox”) request, opioid overdose, psychiatric complaint, or medical complaint.

### Statistical Analysis

We carried out all analyses using SAS 9.4 (SAS Institute, Cary, NC), and *P* < 0.05 was considered statistically significant.^18^ Frequencies and proportions were tabulated for categorical variables, which included gender, race, health insurance, naloxone kit provision, buprenorphine prescription, comorbid DSM-5 diagnosis, and ED chief complaint. We calculated mean and standard deviation for age, which was treated as a continuous variable. Chi-square and Fisher exact tests were used to compare the categorical demographic and medical characteristics of those with vs those without a repeat opioid-related ED visit within 30 days, 90 days, or one year. We used *t*-tests to assess differences in age by outcome status. Identical methods were used for the non-opioid-related ED visit outcomes (at 30 days, 90 days, and one year). Crude and adjusted Poisson models were constructed to estimate changes in the number of total repeat ED visits as well as the associated 95% confidence interval (CI) between those who were prescribed bup/nx and those who were not at the index ED visit for each of the time periods (30 days, 90 days, and one year). Separate models were generated for each outcome. Although no overdispersion in the 30-day model was observed, overdispersion in the 90-day and one-year models was detected and was accounted for by scaling by the deviance. Secondary analyses examined whether the association between bup/nx prescription and total number of repeat ED visits varied based on whether the patient also received a naloxone kit at their initial ED visit. To accomplish this, we included an interaction term between bup/nx prescription and naloxone kit in each of the models. All adjusted models included age and domicile status as covariates.

## RESULTS

This study included 169 OUD patients. Of these, approximately 67.5% were male and 82.8% were White. Most patients did not have health insurance (72.2%), and 27 (15.9%) were homeless (Tables 1, 2). Additionally, over 75% of patients presented to the ED at their initial visit in opioid withdrawal or requesting “detox.” Ninety-one patients (53.8%) received ED-initiated bup/nx (suboxone), and 110 (65.1%) were given a naloxone kit to take home at their initial ED visit. A bup/nx prescription was more likely to be given to patients who presented in opioid withdrawal and/or requested “detox” (63.3% vs 36.7%; *P* < 0.001), but bup/nx prescription did not show significant associations with age, gender, race, insurance status, presence of co-morbid DSM-5 diagnosis, or domicile status. A naloxone kit was more likely to be provided to patients who received bup/nx (97.8% vs 26.9%; *P* < 0.001).

**Table 1.** Comparison of patient characteristics by whether the patient had a repeat opioid-related emergency department visit.

Variables	30-Day repeat ED visit			90-Day repeat ED visit			1-Year repeat ED visit		
	No	Yes	*P*-value	No	Yes	*P*-value	No	Yes	*P*-value
	(n = 137)	(n = 32)		(n = 118)	(n = 51)		(n = 102)	(n = 67)	
Age, mean (SD)	36.5 ± 9.6	37.8 ± 8.0	0.49	36.5 ± 9.6	37.3 ± 8.8	0.62	37.0 ± 10.0	36.4 ± 8.4	0.71
Gender, n (%)									
Female	49 (35.8)	6 (18.8)	0.06	44 (37.3)	11 (21.6)	<0.05^*^	35 (34.3)	20 (29.8)	0.54
Male	88 (64.2)	26 (81.2)		74 (62.7)	40 (78.4)		67 (65.7)	47 (70.2)	
Race, n (%)									
White	113 (83.7)	27 (84.4)	0.79	97 (83.6)	43 (84.3)	0.64	86 (86.0)	13 (19.4)	0.33
Black	22 (16.3)	5 (15.6)		19 (16.4)	8 (15.7)		14 (14.0)	54 (80.6)	
Health Ins, n (%)									
Private	18 (13.1)	2 (6.2)	0.49	15 (12.7)	5 (9.8)	0.86	14 (13.7)	6 (9.0)	0.43
Public	23 (16.8)	4 (12.5)		19 (16.1)	8 (15.7)		18 (17.6)	9 (13.4)	
Self-pay	96 (70.1)	26 (81.3)		84 (71.2)	38 (74.5)		70 (68.6)	52 (77.6)	
Homeless, n (%)									
No	117 (85.4)	25 (78.1)	0.31	103 (87.3)	39 (76.5)	0.08	90 (88.2)	52 (77.6)	0.07
Yes	20 (14.6)	7 (21.9)		15 (12.7)	12 (23.4)		12 (11.8)	15 (22.4)	
Naloxone kit given, n (%)									
No	39 (28.5)	20 (62.5)	<0.001^*^	32 (27.1)	27 (52.9)	0.001^*^	28 (27.4)	31 (46.3)	0.01^*^
Yes	98 (71.5)	12 (37.5)		86 (72.9)	24 (47.1)		74 (72.6)	36 (53.7)	
Buprenorphine Rx, n (%)									
No	58 (42.3)	20 (62.5)	0.04^*^	51 (43.2)	27 (52.9)	0.24	45 (44.1)	33 (49.2)	0.51
Yes	79 (57.7)	12 (37.5)		67 (56.8)	24 (47.1)		57 (55.9)	34 (50.8)	
Comorbid DSM-5 Dx, n (%)									
No	116 (84.7)	28 (87.5)	0.68	98 (83.0)	46 (90.2)	0.23	84 (82.4)	60 (89.6)	0.20
Yes	21 (15.3)	4 (12.5)		20 (17.0)	5 (9.8)		18 (17.6)	7 (10.4)	
ED chief complaint, n (%)									
Opioid WD/detox request	102 (74.4)	26 (81.2)	0.52	88 (74.6)	40 (78.4)	0.23	77 (75.5)	51 (76.1)	0.30
Opioid OD	21 (15.3)	2 (6.2)		19 (16.1)	4 (7.8)		15 (14.7)	8 (11.9)	
Psychiatric complaint	9 (6.6)	2 (6.2)		8 (6.8)	3 (5.9)		8 (7.8)	3 (4.5)	
Medical complaint	5 (3.7)	2 (6.2)		3 (2.5)	4 (7.8)		2 (2.0)	5 (7.5)	

Race information was missing for two patients.

*ED*, emergency department; *Detox*, detoxification; *DSM-5*, Diagnostic and Statistical Manual of Mental Disorders, 5th Edition; *Dx*, diagnosis; *Ins*, insurance; *OD*, overdose; *Rx*, prescription; *WD*, withdrawal.

^*^ Denotes statistical significance where *P* < 0.05.

**Table 2.** Comparison of patient characteristics by whether the patient had a repeat non-opioid-related ED visit.

Variables	30-Day repeat ED visit			90-Day repeat ED visit			1-Year repeat ED visit		
	No	Yes	*P*-value	No	Yes	*P*-value	No	Yes	*P*-value
	(n = 158)	(n = 11)		(n = 146)	(n = 23)		(n = 125)	(n = 44)	
Age, mean (SD)	36.3 ± 9.2	43.8 ± 8.9	0.009^*^	36.0 ± 9.0	41.9 ± 9.8	0.004^*^	35.5 ± 9.1	40.3 ± 9.3	0.003^*^
Gender, n (%)									
Female	53 (33.5)	2 (18.2)	0.51	50 (34.2)	5 (21.7)	0.23	43 (34.4)	12 (27.3)	0.39
Male	105 (66.5)	9 (81.8)		96 (65.8)	18 (78.3)		82 (65.6)	32 (72.7)	
Race, n (%)									
White	130 (83.3)	10 (90.9)	0.75	121 (84.0)	19(82.6)	0.83	103 (83.7)	37 (84.1)	0.70
Black	26 (16.7)	1 (9.1)		23 (16.0)	4 (17.4)		20 (16.3)	7 (15.9)	
Health ins, n (%)									
Private	18 (11.4)	2 (18.2)	0.68	17 (11.6)	3 (13.0)	0.005^*^	15 (12.0)	5 (11.4)	0.36
Public	25 (15.8)	2 (18.2)		18 (12.3)	9 (39.1)		17 (13.6)	10 (22.7)	
Self-pay	115 (72.8)	7 (63.6)		111 (76.0)	11 (47.8)		93 (74.4)	29 (65.9)	
Homeless, n (%)									
No	134 (84.8)	8 (72.7)	0.39	127 (87.0)	15 (65.2)	0.008^*^	112 (89.6)	30 (68.2)	0.001^*^
Yes	24 (15.2)	3 (27.3)		19 (13.0)	8 (34.8)		13 (10.4)	14 (31.8)	
Naloxone kit given, n (%)									
No	55 (34.8)	4 (36.4)	0.92	46 (31.5)	13 (56.5)	0.02^*^	41 (32.8)	18 (40.9)	0.33
Yes	103 (65.2)	7 (63.6)		100 (68.5)	10 (43.5)		84 (67.2)	26 (59.1)	
Buprenorphine Rx, n (%)									
No	73 (46.2)	5 (45.4)	0.96	64 (43.8)	14 (60.9)	0.13	58 (46.4)	20 (45.4)	0.91
Yes	85 (53.8)	6 (54.6)		82 (56.2)	9 (39.1)		67 (53.6)	24 (54.6)	
Comorbid DSM-5 Dx, n (%)									
No	134 (84.8)	10 (90.9)	0.58	123 (84.2)	21(91.3)	0.53	108 (86.4)	36 (81.8)	0.46
Yes	24 (15.2)	1 (9.1)		23 (15.8)	2 (8.7)		17 (13.6)	8 (18.2)	
ED chief complaint, n (%)									
Opioid WD /detox request	118 (74.7)	10 (90.9)	0.62	109 (74.7)	19 (82.6)	0.29	93 (74.4)	35 (79.6)	0.32
Opioid OD	22 (13.9)	1 (9.1)		21 (14.4)	2 (8.7)		20 (16.0)	3 (6.8)	
Psychiatric complaint	11 (7.0)	0 (0.0)		11 (7.5)	0 (0.0)		8 (6.4)	3 (6.8)	
Medical complaint	7 (4.4)	0 (0.0)		5 (2.4)	2 (8.7)		4 (3.2)	3 (6.8)	

Race information was missing for two patients.

*ED*, emergency department; *Detox*, detoxification; *DSM-5*, Diagnostic and Statistical Manual of Mental Disorders, 5th Edition; *Dx*, diagnosis; *Ins*, insurance; *OD*, overdose; *Rx*, prescription; *WD*, withdrawal.

^*^ Denotes statistical significance where *P* < 0.05.

At 30 days, 32 patients (18.9%) had a repeat opioid-related ED visit (Table 1). No significant differences emerged in terms of age, gender, race, health insurance status, homelessness, ED chief complaint, or comorbid DSM-5 diagnosis rates. However, bup/nx prescription and naloxone kit provision were associated with decreased ED utilization for opioid-related visits at 30 days (*P* = 0.04 and *P* < 0.001, respectively). By 90 days, 30.2% of the study sample had a repeat opioid-related ED visit. In this time frame, male patients (*P* < 0.05) and those who did not receive a naloxone kit (*P* = 0.001) were more likely to have a repeat visit; however, ED-prescribed bup/nx was no longer significantly associated with having a repeat visit (*P* = 0.24).

Within one year, 67 patients (40.0%) had a repeat opioid-related ED visit. In this time frame, the only variable showing a significant association with repeat ED visit was naloxone kit provision (*P* = 0.01). Of those who received a naloxone kit, 32.7% had a repeat visit; however, among those who did not receive a kit, 52.5 % had a repeat visit. Thus, naloxone kit provision was associated with decreased ED utilization for opioid-related visits at 30 days, 90 days, and one year (*P* < 0.001, *P* = 0.001, and *P* = 0.01, respectively). Of the 169 patients, only 11 (6.5%) had a non-opioid-related repeat ED visit within 30 days (Table 2), compared with 32 (18.9%) who had an opioid-related repeat ED visit in that same time frame. Increasing age was associated with a repeat non-opioid-related visit at 30 days (43.8 ± 8.9 years vs 36.3 ± 9.2 years; *P* = 0.009). At this time point, no significant differences emerged in terms of gender, race, health insurance, homelessness, naloxone kit provision, bup/nx prescription, comorbid DSM-5 diagnosis, or ED chief complaint.

By 90 days, the number of patients with a non-opioid-related repeat ED visit increased to 23 (13.6%). Those with a repeat visit were older (*P* = 0.004), more likely to be uninsured (*P* = 0.005), more likely to be homeless (*P* = 0.008), and less likely to have received a naloxone kit at the initial visit (*P* = 0.02). By one year, 44 patients (26%) had a repeat non-opioid-related ED visit. Again, patients with a repeat visit were older (*P* = 0.003) and more likely to be homeless (*P* < 0.001), although insurance status and naloxone provision no longer showed a significant association (*P* = 0.36).

Next, the total repeat all-cause ED visits were considered. Within 30 days of their index ED visit, 23.1% of patients had at least one repeat all-cause ED visit (range 1–4 visits). By 90 days, this percentage increased to 35.5% (range 1–12 visits). At one year from the initial visit, 50.3% of patients had a repeat visit (range 1–36 visits). In the unadjusted models, bup/nx prescription provision was significantly associated with a reduction in the number of repeat all-cause ED visits at 90 days (but not 30 days or one year) (Table 3). Given that significant association was also observed between older age and homelessness and all-cause repeat ED visits, the bup/nx association findings were re-evaluated after adjusting for age and domicile status. After adjusting for age and domicile status, a stronger association emerged between bup/nx prescription provision and repeat all-cause ED visits, with bup/nx prescription being associated with a 44% reduction in the number of repeat all-cause ED visits at 30 days (adjusted incidence rate ratio [IRR]:0.56, 95% confidence interval [CI] 0.33–0.96), a 57% reduction at 90 days (adjusted IRR 0.43, 95% CI 0.27–0.69), and a 40% reduction at one year (adjusted IRR 0.60, 95% CI 0.39–0.92) (Table 3).

**Table 3.** Count ratios and 95% confidence intervals for the association between buprenorphine/naloxone prescription given and number of all-cause repeat emergency department visits.^+^

	Repeat ED visit within 30 days		Repeat ED visit within 90 days		Repeat ED visit within 1 year	
	Crude (95% CI)	Adjusted^1^ (95% CI)	Crude (95% CI)	Adjusted^1^ (95% CI)	Crude (95% CI)	Adjusted^1^ (95% CI)
Overall						
No bup/nx	Ref	Ref	Ref	Ref	Ref	Ref
Bup/nx given	0.60 (0.35–1.02)	**0.56** **(0.33–0.96)**	**0.48** **(0.29–0.79)**	**0.43** **(0.27–0.69)**	0.66 (0.42–1.05)	**0.60** **(0.39–0.92)**
No naloxone kit given						
No bup/nx	Ref	Ref	Ref	Ref	Ref	Ref
Bup/nx given	0.95 (0.13–6.97)	1.10 (0.15–8.13)	0.37 (0.03–4.88)	0.50 (0.04–5.68)	0.39 (0.03–5.66)	0.52 (0.04–6.54)
Naloxone kit given						
No bup/nx	Ref	Ref	Ref	Ref	Ref	Ref
Bup/nx given	1.73 (0.52–5.78)	1.50 (0.45–5.07)	3.46 (0.75–15.97)	2.67 (0.63–11.28)	2.38 (0.76–7.44)	1.85 (0.63–5.44)

^+^ Estimates of count ratio and 95% CIs generated from Poisson models.

^*^ **Bold face font** indicates statistical significance where *P* < 0.05.

^1^ Adjusted for age and domicile status.

*ED*, emergency department; *CI*, confidence interval; *bup/nx*, buprenorphine/naloxone; *ref*, reference.

## DISCUSSION

This study highlights the impact of OUD and the opioid epidemic in general on the ED. Over half the patients included in this study had a repeat ED visit within one year. This high level of utilization is likely due, in large part, to the overlapping social risk and social need experienced by this cohort. The general demographic characteristics of this study population are similar to the national opioid epidemic landscape, predominantly White (82.8%) and male (67.5%).^19^ However, when considering social factors, such as insurance and domicile status, our OUD population was disproportionately affected by negative social determinants of health (SDoH). More than seven in ten OUD patients were uninsured, compared with the average uninsured rate of 12.7% in non-expansion states in 2021.^20^ Further, 16% were homeless, which is nearly 100 times the national rate.^21^ Homelessness and lack of insurance were independently associated with increased ED utilization for non-opioid-related visits at 90 days (*P* = 0.008 and p = 0.005, respectively), and again at one year for homelessness (*P* < 0.001). This underscores the complex social context of the ED OUD population. If co-occurring SDoH domains are not addressed during the ED visit, MOUD may not be successful in decreasing subsequent healthcare utilization. At UAB Hospital, ED social workers and case managers are available 24/7 to provide housing and healthcare access resources to underserved patients; however, referrals to assistance programs are not consistently documented in the EHR.

Although bup/nx provision was associated with decreased ED utilization for opioid-related visits at 30 days (*P* = 0.04), only 53.8% received ED-initiated bup/nx. Further, bup/nx was more likely to be given to OUD patients who presented in opioid withdrawal and/or requesting “detox” (63.3% vs 36.7%; *P* < 0.001). There are many plausible explanations for why 46.2% of OUD patients did not receive bup/nx at the initial ED visit, although this percentage is much lower than a recently published national retrospective cohort study where 91.5% were not prescribed buprenorphine after an ED visit for opioid overdose.^22^ First, in July 2019 (study period start date), the UAB Department of Emergency Medicine had just initiated the Drug Addiction Treatment Act of 2000 (DATA 2000) “X-waiver” training requirement to license emergency clinicians for MOUD prescribing bup/nx through an incentive program, which was strongly encouraged but not mandated for all clinicians.^23^

Further, MOUD program uptake was not universal due to several known barriers to MOUD in the ED, including lack of training and experience in SBIRT, lack of availability of close outpatient follow-up in addiction treatment centers, and limited clinician time in a busy ED.^24^ Finally, not every OUD patient presenting to the ED was a candidate for MOUD with bup/nx due to lack of motivation to seek and engage in outpatient treatment, concomitant use of illicit depressive agents, hypersensitivity reaction, and concern for diversion.^25^ It is standard practice at the UAB ED for patients receiving ED-initiated MOUD to be referred to community treatment programs; however, outpatient follow-up rates are not easily measured within our current system.

While roughly half of the patients received MOUD at the initial ED visit, nearly two-thirds received a take-home naloxone kit, which, at the time of the study was provided to patients free of charge with an emergency physician (EP) order via a collaborative project with the Jefferson County Health Department. Importantly, naloxone kit provision was associated with decreased ED utilization for opioid-related visits at 30 days, 90 days, and one year (*P* < 0.001, *P* = 0.001, and *P* = 0.01, respectively) and non-opioid-related visits at 90 days (*P* = 0.02). Naloxone is a potentially life-saving, easy-to-use and, in this instance, free intervention. Several factors might have contributed to incomplete provision: 1) The naloxone kit required a specific EP order to be dispensed, which may not have been prioritized due to competing demands for physician focus and time; 2) EPs may have had misperceptions of time-consuming counseling accompanying naloxone provision; and 3) EPs may have been unaware of the availability of naloxone provided as a take-home kit rather than a prescription.

In general, there was significant collinearity between bup/nx and naloxone kit provision. A naloxone kit was more likely to be provided to patients who received bup/nx (97.8% vs 26.9%; *P* < 0.001). Further, bup/nx was more likely to be given to patients who presented in opioid withdrawal and/or requested “detox: (63.3% vs 36.7%; *P* < 0.001). However, patients who presented in the most severe form of OUD, an acute overdose, were not more likely to receive bup/nx. This may be due to the EP’s focus on resuscitation of acute decompensation and respiratory depression, rather than engagement of a brief intervention for MOUD to assess a patient’s motivation toward behavioral change.

Our study is unique in assessing whether ED-initiated bup/nx impacts subsequent acute healthcare utilization, while also evaluating the impact of SDoH, such as health insurance and domicile status. Our results showed that when controlling for age and homelessness, initiation of bup/nx in the ED setting was associated with decreased subsequent all-cause ED utilization. Further, socioeconomic factors, specifically insurance and domicile status, appear to have significant impact on non-opioid-related ED reuse. These findings demonstrate the ED’s potential as an initiation point for OUD treatment and highlight the importance of considering social risk and social need for OUD patients in the ED.

## LIMITATIONS

This study had several limitations. First, the study design was a retrospective chart review, which prevents abstractors from being blinded to the study purpose and drawing conclusions of causality. However, to minimize bias, established emergency medicine chart review study methods were adhered to.^26^ Further, the study population was obtained from a single site, which limits generalizability. Revisits to EDs in outside healthcare systems were unable to be tracked, preventing complete capture. However, UAB Hospital is the catchment healthcare system for the state of Alabama providing healthcare access to underserved populations, including the Charity Care Program, Equal Access Birmingham free clinic, Providing Access to Healthcare clinic, and a Comprehensive Urban Underserved and Rural Experience program. Finally, ED visit rates for opioid overdose increased by over 25% in 2020 due to the COVID-19 pandemic, despite a decline in overall ED visits.^27^ Thus, expanded community- and hospital-based MOUD interventions were needed to support OUD patients during the COVID-19 pandemic; however, many counseling and treatment clinics were unavailable during that time.

## CONCLUSION

Initiation of buprenorphine/naloxone in the ED setting can result in decreased subsequent ED utilization. Socioeconomic factors, specifically health insurance and domicile status, also appear to have a significant impact on ED reuse. These findings demonstrate the ED’s potential as an initiation point for prescribing medication for opioid use disorder and highlight the importance of considering social risk and social need for OUD patients.
